# Replication of the Venezuelan Equine Encephalitis Vaccine from a Synthetic PCR Fragment

**DOI:** 10.3390/pharmaceutics16091217

**Published:** 2024-09-17

**Authors:** Christine Mathew, Colin Tucker, Irina Tretyakova, Peter Pushko

**Affiliations:** Medigen, Inc., Frederick, MD 21701, USA

**Keywords:** VEE virus, DNA vaccine, synthetic vaccine

## Abstract

Background/Objectives: There is no approved human vaccine for Venezuelan equine encephalitis (VEE), a life-threatening disease caused by the VEE virus (VEEV). In previous studies, plasmid DNA encoding the full-length RNA genome of the VEE V4020 vaccine was used for the preparation of experimental live virus VEE vaccines in the plasmid-transfected cell culture. Methods: Here, we used the high-fidelity polymerase chain reaction (PCR) to prepare synthetic, transcriptionally active PCR (TAP) fragments encoding the V4020 genome. Results: TAP fragment initiated the replication of the V4020 live virus vaccine in TAP fragment-transfected cells. A transfection of less than 1 ug of TAP fragment resulted in the replication of the V4020 vaccine virus in CHO cells. Conclusion: We conclude that not only plasmid DNA but also synthetic PCR-generated DNA fragments can be used for the manufacturing of live vaccines for VEEV and, potentially, other viruses.

## 1. Introduction

Venezuelan equine encephalitis (VEE) is a life-threatening disease in humans and animals caused by the VEE virus (VEEV), an alphavirus transmitted by mosquitoes. VEEV is a positive-sense, enveloped virus approximately 70 nm in diameter, with a single-stranded RNA genome of 11.5 kb in length [1,2,3]. In humans, VEEV causes a biphasic febrile illness followed by myeloencephalitis with high morbidity, including neurological complications and a mortality rate of approximately 2% [4]. The similarity of the initial symptoms of VEE, such as fever and headache, with other infections complicates clinical diagnosis. In addition, considerable overlap exists between geographic regions of VEEV endemicity and those of some other tropical diseases. In particular, it has been reported that an overlap with areas of dengue virus infection may result in an underestimation of VEEV prevalence [5]. Ecological and climate changes may be contributing factors to the increased geographical distribution of *Culex* mosquitos, increasing the population of humans and equines at risk for contracting VEEV [6]. VEEV is also recognized as a potential biological weapon, due to the severe threat posed to human and animal health and the minimal infectious aerosol dose required for infection. Finally, VEEV has a history of being implicated in laboratory-acquired infections [7,8,9]. Taken together, these factors suggest the continuing possibility of VEEV outbreaks, including in non-endemic areas.

Currently, there is no approved human vaccine for VEEV. Experimental live virus vaccines and DNA vaccines have been developed for parenteral administration [10,11,12]. Plasmid DNA was also used as an infectious clone to prepare novel VEE vaccines [13,14]. Previously, we developed an experimental live-attenuated V4020 vaccine virus by rearranging the VEEV genome and introducing additional attenuating mutations [3,11,15]. The V4020 vaccine virus was prepared by using the infectious clone pMG4020 encoding the whole RNA genome of the V4020 virus under transcriptional control of the cytomegalovirus (CMV) promoter. This technology, sometimes called iDNA, allowed the preparation of the live virus vaccine V4020 in the cells transfected with pMG4020 plasmid rather than that infected with the V4020 seed virus. Therefore, plasmid DNA can be potentially used as a vaccine seed, which offers many advantages, compared to a traditional virus seed, such as genetic and thermal stability, storage, and transportation of DNA [3,16]. DNA is easy to manufacture, and only 10–100 ng of plasmid was shown to launch the vaccine virus [11,17], which can improve the manufacturing and scale-up of vaccine production during outbreaks. The proven ability of plasmid DNA to launch a live vaccine virus in plasmid-transfected cells prompted us to explore whether synthetic DNA generated by polymerase chain reaction (PCR) can also initiate the replication of the live-attenuated vaccine virus in cell culture. The objective of this study was to evaluate if the PCR fragment containing the full genome of the VEE vaccine can be used for the preparation of the live-attenuated VEE vaccine in the cell culture. If successful, the use of PCR can facilitate VEE vaccine scale-up manufacturing and outbreak response. Here, we report that a synthetic, transcriptionally active PCR (TAP) fragment encompassing the full-length cDNA genome of the V4020 virus downstream from the CMV promoter can launch the replication of the live-attenuated VEE vaccine virus in transfected cells.

## 2. Materials and Methods

### 2.1. Cell Culture

Chinese hamster ovary (CHO) and Vero cell lines were obtained from the American Type Culture Collection (Manassas, VA, USA) and maintained in a humidified incubator at 37 °C in 5% CO_2_ in MEMα supplemented with 10% fetal bovine serum (FBS) and gentamicin sulfate (50 µg/mL) (Life Technologies, Carlsbad, CA, USA).

### 2.2. Plasmids and PCR

Plasmid pMG4020 encoding rearranged V4020 VEEV RNA was described elsewhere [11,15]. Briefly, pMG4020 encoded the full-length, rearranged genome of the V4020 live-attenuated VEEV vaccine downstream from the cytomegalovirus (CMV) immediate-early enhancer and promoter. Another plasmid, pMG4023, was prepared by digesting pMG4020 at a unique SalI site, by Q5 polymerase treatment, and by re-ligation using the T4 Quick ligation kit (New England Biolabs). This resulted in the addition of 4 bp to the V4020 nsP1 gene, which caused a frameshift mutation intended to prevent the formation of live V4020. For all transcriptionally active PCR fragments, the CMV promoter sequence was included upstream from the V4020 5′ RNA transcription start site. Plasmid sequences were confirmed by whole plasmid sequencing using the Oxford Nanopore Technologies instrument (Poochon Scientific, Frederick, MD, USA). PCR fragments of approximately 12.5 kb in length encompassing the CMV promoter and the full-length cDNA of V4020 were generated by using high-fidelity Q5 polymerase (New England Biolabs, Ipswich, MA, USA) according to the manufacturer’s instructions, with specific oligonucleotide primers. Briefly, 50 ng of pMG4020 was used as a template in a 25 µL PCR reaction, including oligonucleotides 5′- GAATTCGGCGCGCCTGACATTGATTATTG and 5′- GCCGACCCAGTAGGCCGCCCTTTTTTTTTTTTTT as forward and reverse primers, respectively. The PCR fragment was purified using the PCR clean-up system (Promega, Fitchburg, WI, USA) and treated with DpnI to remove template pMG4020. After DpnI treatment, the PCR fragment was purified again with the PCR clean-up system and eluted in 50 µL of DNAse-free sterile water.

Next, we prepared synthetic PCR fragments encoding the V4020 virus using pMG4023 as a template. The latter plasmid was prepared by a SalI digest of pMG4020, by treatment with Q5 polymerase, and by re-ligation. This resulted in a lethal frameshift mutation within the nsP1 gene of the V4020 genome (Supplementary Figure S1). To restore the full-length V4020 sequence by PCR, we initially prepared the 2.2 kb and 10.3 kb PCR fragments using oligonucleotide primers designed to repair a frameshift SalI mutation and then ligated these fragments to restore the full-length V4020 genome. Briefly, the 2.2 kb fragment was prepared using primers 5′- GAATTCGGCGCGCCTGACATTGATTATTG and 5′- GGCCCCAGCCTCTTGTAACATCAAGTCGACATCGGCTTCCAGAGTGGGCTCCTCAAC (SalI site is underlined) and included the CMV promoter and V4023 sequence, including the repaired SalI site. The 10.3 kb fragment was generated using primers 5′- GTTGAGGAGCCCACTCTGGAAGCCGATGTCGACTTGATGTTACAAGAGGCTGGGGCC (SalI site is underlined) and 5′- GCCGACCCAGTAGGCCGCCCTTTTTTTTTTTTTT and encompassed the sequence starting from the repaired SalI site to the 3′ terminus of the V4023 sequence (Supplementary Figure S1). Then, the 2.2 kb and 10.3 kb PCR fragments were digested with SalI and ligated in vitro (QuickLigase kit, New England Biolabs, MA, USA) to generate a 12.5 kb synthetic, transcriptionally active PCR fragment encoding the V4020 vaccine virus with repaired frameshift mutation in V4023 nsP1. The synthetic, ligated PCR fragment was purified as described above.

### 2.3. Transfections and Assays

CHO cells were transfected with 15–30 µL (0.2–0.5 µg) of the final purified PCR fragments by electroporation of DNA in 75 cm^2^ flasks. Transfection was performed essentially as described previously [18]. Alternatively, transfection was performed by using the Fugene 6 transfection reagent according to the manufacturer’s instructions (Promega). As controls, pMG4020 (positive control) and pMG4023 (negative control) plasmids were used in transfections, as well as in non-transfected cells. The vaccine virus was propagated in transfected CHO cells in 75 cm^2^ flasks. At 48 h post-infection, the virus-containing supernatant was harvested, clarified, and frozen at −80 °C in 1 mL aliquots.

To confirm the presence of the virus in transfected cells, immediately after electroporation, a 0.4 mL aliquot of transfected CHO cells was seeded and grown for 24 h in 8-well chamber slides in MEMα containing 10% FBS, followed by IFA, as described below. Alternatively, the vaccine virus in the supernatant of transfected cells was used to infect freshly grown CHO cells in the chamber slides. Transfection supernatant samples were diluted at 100-fold increments and absorbed (0.1 mL/well) onto CHO cell monolayers for 1 h at 37 °C. Then, 0.3 mL of the αMEM medium was added per well, and incubation was continued for 24 h. For IFA, cells were fixed with cold acetone at −20 °C for 20 min and probed with primary rabbit antisera, followed by fluorescein-labeled secondary antibody to rabbit IgG (H&L) (Seracare, Gaithersburg, MD, USA). Primary rabbit antiserum was raised in a rabbit immunized with the V4020 vaccine, as described elsewhere [12].

For Western blot, the transfected CHO cells were harvested using a cell scraper, and V4020 vaccine proteins were separated using 4–12% gradient SDS-PAGE (Genscript, Piscataway, NJ, USA) and probed with anti-V4020 rabbit antisera. For detection, alkaline phosphatase-labeled secondary antibody to rabbit IgG (H&L) (Seracare) was used.

For virus titer determination, virus samples were harvested after 24–48 h post-transfection and quantitated in duplicates using a standard plaque assay in Vero cell monolayers.

## 3. Results

### 3.1. Preparation of Transcriptionally Active PCR (TAP) Encoding the V4020 Vaccine Virus

**Figure 1:**
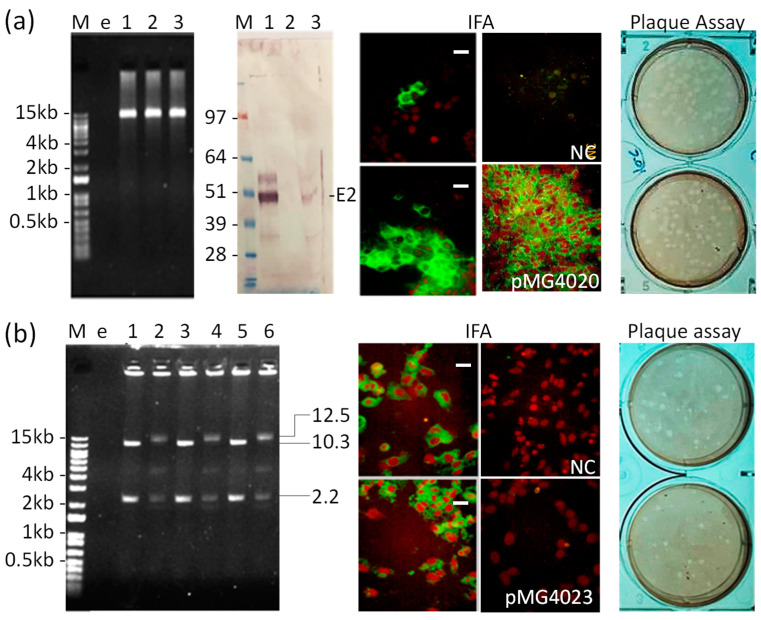
Preparation of the live-attenuated VEE virus vaccine by the transfection of CHO cells with transcriptionally active PCR (TAP) fragments. (**a**) **Left panel**, 12.5 kb TAP fragments (lanes 1, 2, and 3), prepared by high-fidelity PCR using pMG4020 as a template in ethidium bromide-stained 1% agarose/TAE gel. M, 1 kb Plus DNA ladder (Thermofisher, Waltham, MA, USA), with band size indicated on the left; e, empty lane. **Middle panel**, Western blot of CHO cells transfected with pMG4020 (lane 1) or TAP fragment (lane 3). Cells were harvested 48 h post-transfection and probed with polyclonal antibody to VEEV. The major band consistent with the E2 antigen is indicated. M, SeeBlue Plus2 protein ladder (Thermofisher); e, empty lane. **Right panel**, detection of V4020 antigen expression by IFA (green color; Bar, 50 µm) in CHO cells transfected with a 12.5 kb TAP fragment, PBS (negative control, NC), and pMG4020 (positive control), as well as the detection of the V4020 virus from the supernatant of transfected CHO cells by plaque assay in Vero cells. (**b**) **Left panel**, preparation of 2.2 kb and 10.3 kb TAP fragments in triplicate using a mutant pMG4023 plasmid as a template (lanes 1, 3, and 5). Preparation of a 12.5 kb synthetic PCR fragment by in vitro ligation is also shown (lanes 2, 4, and 6) in triplicate. **Right panel**, detection of V4020 antigen expression (green color; Bar, 50 µm) by IFA in CHO cells transfected with a 12.5 kb synthetic PCR fragment, PBS (NC), and pMG4023, as well as the detection of the V4020 virus from the supernatant of transfected cells by plaque assay in Vero cells. See Supplementary Figure S1 for more details.

The plasmid pMG4020 encoding the full-length, rearranged RNA genome of V4020 downstream from the CMV promoter was previously shown to initiate the replication of the V4020 vaccine virus in vitro and in vivo [11,12]. Here we investigated whether the synthetic PCR fragment can be used to start the replication of V4020 in vitro. In the first set of experiments, we prepared a transcriptionally active PCR (TAP) fragment encompassing the full-length cDNA of the V4020 VEEV vaccine virus downstream from the CMV promoter. To reduce the possibility of PCR-derived mutations, PCR conditions were optimized, and long-range PCR was conducted using high-fidelity Q5 DNA polymerase. As a result, a 12.5 kb PCR fragment was generated (Figure 1). The PCR fragment was confirmed by 1% agarose gel, restriction digest, OD_260_, and DNA sequencing. To evaluate whether the PCR fragment launched the V4020 vaccine virus, the PCR fragment was treated with DpnI to remove template plasmid DNA pMG4020 and transfected into CHO cells. The DpnI restriction enzyme is specific for methylated bacterial DNA and was used to remove template DNA from PCR reactions. As controls, we used pMG4020- and negative control (PBS or water)-transfected cells. In the transfected cells, the CMV promoter was designed to initiate the run-off transcription of the full-length genomic RNA in transfected cells in situ. Expression was confirmed by IFA (Figure 1a) in both PCR- and control pMG4020-transfected cells. Additionally, Western blot indicated a band of the VEEV E2 expected size in the PCR-transfected cells (lane 3), although the band was not as apparent as that in pMG4020-transfeted cells (lane 1). Finally, the V4020 vaccine virus was confirmed by plaque assay (Figure 1a) in the medium from transfected CHO cells, with titers ranging from 10^7^–10^8^ PFU/mL.

To investigate if V4020 was launched by the PCR fragment and not the residual pMG4020 plasmid template in the PCR reaction, we transformed the DpnI-treated PCR fragment into *E. coli* to detect if any residual intact template pMG4020 was present in the DpnI-treated PCR reaction. As a control, we used an aliquot of the PCR fragment that was not treated with DpnI. In both cases (DpnI-treated and untreated PCR fragment), *E. coli* colonies were detected, although the DpnI-treated fragment produced approximately 50% fewer colonies, compared to the DpnI-untreated sample (Supplementary Figure S2a). Ten colonies from each bacterial plate were grown, and plasmids were isolated and analyzed by restriction digest to evaluate if the intact pMG4020 was present. In the DpnI-treated sample, only 4 out of 10 colonies contained intact pMG4020 while others contained various deletion variants of the template pMG4020. In contrast, 9 out of 10 colonies in the DpnI-untreated sample represented intact pMG4020 (Supplementary Figure S2b). In other experiments, the template plasmid was further degraded by digesting the PCR preparation with restriction enzymes within the plasmid template but outside of the PCR fragment, by treatment with the S1 and mung bean nucleases specific for single-stranded DNA and RNA, by the transformation of the treated samples into *E. coli*, and by DNA sequencing Taken together, data suggested that treatment with DpnI and other enzymes reduced the amount of the pMG4020 template to less than 0.2% (<0.1 ng) of the original concentration present in the PCR reaction, which is below the lowest amount of plasmid DNA shown to launch the vaccine virus [11,12]. These results suggested that the PCR fragment launched the replication of the V4020 vaccine virus in transfected CHO cells.

### 3.2. Preparation of Synthetic PCR Fragment Using Ligation In Vitro

To confirm the launch of the V4020 virus from the TAP fragment and to exclude any role of the residual pMG4020 template plasmid in launching the V4020 vaccine virus, we prepared a pMG4023 plasmid containing a frameshift mutation within the carboxy terminal part of the nsP1 gene of V4020. Then, PCR was conducted using pMG4023 and oligonucleotide primers designed to repair the frameshift mutation. Initially, two PCR (2.2 kb and 10.3 kb) fragments were prepared (Figure 1b), each containing the repaired mutation, with the intent to conduct an overlapping PCR to generate the full-length 12.5 kb TAP fragment with a restored nsP1 reading frame (Supplementary Figure S1b). However, attempts to prepare a single PCR fragment using overlapping PCR were unsuccessful. Therefore, the 2.2 kb and 10.3 kb PCR fragments were digested with SalI and ligated in vitro to prepare a synthetic, transcriptionally active, full-length DNA fragment. The ligation in vitro was performed in triplicate and allowed to generate the quantity of the full-length 12.5 kb DNA sufficient for transfection (Figure 1b). The result of in vitro ligations confirmed the presence of the expected new band, which was ~12.5 kb in length, while quantities of 2.2 kb and 10.3 kb were reduced after ligation. An additional band of ~4.4 kb was detected, which likely represented a dimer of the 2.2 kb PCR fragment. Finally, high-molecular-weight material was also observed on the 1% agarose gel, which largely remained within the gel loading wells (Figure 1b).

To evaluate whether the synthetic DNA fragment launched the V4020 vaccine virus, ligation reaction was transfected into CHO cells. As a control, a non-ligated mixture of 2.2 kb and 10.3 kb was included. Controls also included pMG4020-, pMG4023-, and negative control (PBS)-transfected cells. The expression was confirmed by IFA in ligation-transfected cells (Figure 1b). The presence of the V4020 vaccine virus in the supernatant of transfected cells was confirmed by plaque assay (Figure 1b). As expected, the transfection of CHO cells with control pMG4020 also resulted in the expression of the V4020 virus (Figure 1a). Neither pMG4023 nor the negative control resulted in a detectable V4020 virus or antigens by plaque assay or IFA, respectively (Figure 1).

## 4. Discussion

The focus of this short communication is the feasibility of a synthetic PCR fragment to initiate the replication of the live virus VEE vaccine in cell culture. Previously, infectious clone plasmids were used for the rational design of experimental live-attenuated VEEV vaccines [13,14]. Live virus vaccines have the advantage of eliciting rapid, long-lasting immunity with a single vaccination, which is important when rapid containment of disease outbreaks is needed, such as with VEEV. We previously described the VEEV iDNA infectious clone designed to launch live-attenuated VEEV in transfected cells from a plasmid, without the in vitro transcription step [17]. The live-attenuated V4020 vaccine virus was prepared by rearranging the VEEV genome and introducing attenuating mutations in the pMG4020 plasmid [11]. The whole RNA genome of the V4020 virus was placed downstream of the CMV promoter. After plasmid transfection, genomic RNA was synthesized intracellularly, launching the replication of the V4020 vaccine in transfected cells [11,17]. In this study, we evaluated another application of iDNA technology by preparing a transcriptionally active PCR fragment, TAP, encompassing the CMV promoter and the full-length cDNA of the V4020 VEEV vaccine. The synthetic DNA fragment of 12.5 kb in length was generated using either high-fidelity PCR or by the ligation of PCR fragments in vitro. Notably, ligation in vitro was also used to prepare the first infectious clone for the yellow fever virus [19]. We showed that when transfected into CHO cells, a synthetic TAP fragment successfully launched the V4020 vaccine virus, suggesting that a PCR fragment can be used in place of a virus or a pMG4020 plasmid for VEEV vaccine manufacturing. Potentially, this can be useful in situations when a virus seed or bacterial-based plasmid production is not available for vaccine production, for example, when a rapid change in vaccine is needed due to an evolving pathogen. TAP DNA is thermally stable and can improve the time needed for vaccine preparation, which would reduce VEEV-associated morbidity and mortality. The challenges are that plasmid DNA is still required as a template for PCR, and the optimization of long-range PCR is needed to prepare the full-length, functional TAP PCR fragment.

TAP fragments encoding reporter genes were described in earlier studies as a rapid and convenient way to express proteins to decipher the function of putative genes identified by worldwide genomic sequencing projects [20]. For example, this method was applied for an in vitro humoral immunoscreening of novel target antigens from the *Plasmodium* parasite [21]. In another study, this method allowed for a rapid functional assay without the need to construct expression vectors [22]. Our research suggests that the TAP approach can also be applicable for the rapid preparation of live-attenuated virus vaccines without the need to prepare the vaccine virus seed.

The immunogenicity of transcriptionally active PCR fragments in vivo was reported. For example, the immune responses of such PCR fragments encoding a subset of the model *P. falciparum* and *P. yoelii* antigens in mice were equivalent or superior to those induced by the corresponding plasmid DNA vaccines [21]. We described elsewhere that the VEEV iDNA plasmid pMG4020 induced an immune response and protection against VEEV when injected in vivo [11,12]. Potentially, the TAP fragment can be used for in vivo vaccination as well. One limitation of this communication is that TAP was not tested in vivo. However, we planned this experiment separately, as the immunogenicity of TAP fragments encoding live virus vaccines still needs to be tested in vivo. In case the live V4020 vaccine is launched from TAP in vivo, it is expected that an immune response is elicited by the live V4020 virus vaccine, including virus neutralizing and cell-mediated immunity. Unlike standard plasmid DNA vaccines and mRNA vaccines that deliver subunit antigens in vivo, TAP serves as a delivery vehicle for the live virus vaccine, containing multiple vaccine-relevant antigens. It should be noted that PCR fragments may require a higher dose, compared to plasmid DNA, to launch a live virus in vivo, as it has been reported that linear DNA has a lower transfection efficiency, compared to supercoiled plasmid DNA [23]. If successful in vivo, a transcriptionally active PCR fragment can offer unique advantages, compared to standard DNA and mRNA vaccine platforms [24].

## Data Availability

The datasets presented in this study can be found in the article/Supplementary Material and are available upon request.

## Supplementary Materials

The following supporting information can be downloaded at: https://www.mdpi.com/article/10.3390/pharmaceutics16091217/s1, Figure S1. Schematic design for preparation of transcriptionally active PCR (TAP) fragments encoding live virus V4020 vaccine. (a) High-fidelity, long-range PCR using pMG4020 infectious clone as a template. CMV promoter region (CMV) and the full-length genome of V4020 vaccine virus (V4020) are indicated. (b) Preparation of synthetic TAP fragment using the frameshift mutant pMG4023 as a template and ligation of PCR fragments in vitro. Location of frameshift mutation is indicated with X in red. Figure is not at scale. Figure S2. Characterization of TAP fragment after high-fidelity, long-range PCR and treatment with DpnI. (a) TAP fragment, untreated with DpnI (left) and treated with DpnI (right) was transformed into Stbl3 *E. coli* cells to compare the number of colonies resulting from residual pMG4020 plasmid template in a high-fidelity PCR reaction. (b) Plasmid DNA from ten colonies from each plate were isolated and digested with NruI and EcoRV restriction enzymes to identify template pMG4020 plasmid. Expected sizes for pMG4020 are 13 kb and 2 kb indicated on the right.
