# Supplementary material for: Replication of the Venezuelan Equine Encephalitis Vaccine from a Synthetic PCR Fragment
Source: Pharmaceutics. 2024 Sep 17;16(9):1217. doi: 10.3390/pharmaceutics16091217 (PMC11434715; doi:10.3390/pharmaceutics16091217)
Supplement: Supplementary file 1 [file pharmaceutics-16-01217-s001.zip › pharmaceutics-3164061-supplementary.pdf]

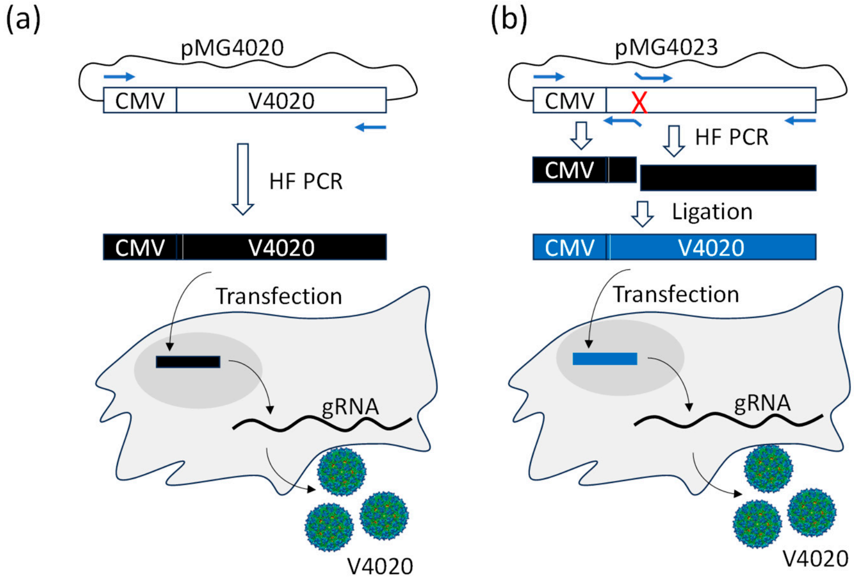


**Figure S1.** Schematic design for preparation of transcriptionally active PCR (TAP) fragments encoding live virus V4020 vaccine. (a) High-fidelity, long-range PCR using pMG4020 infectious clone as a template. CMV promoter region (CMV) and the full-length genome of V4020 vaccine virus (V4020) are indicated. (b) Preparation of synthetic TAP fragment using the frameshift mutant pMG4023 as a template and ligation of PCR fragments in vitro. Location of frameshift mutation is indicated with X in red. Figure is not at scale.

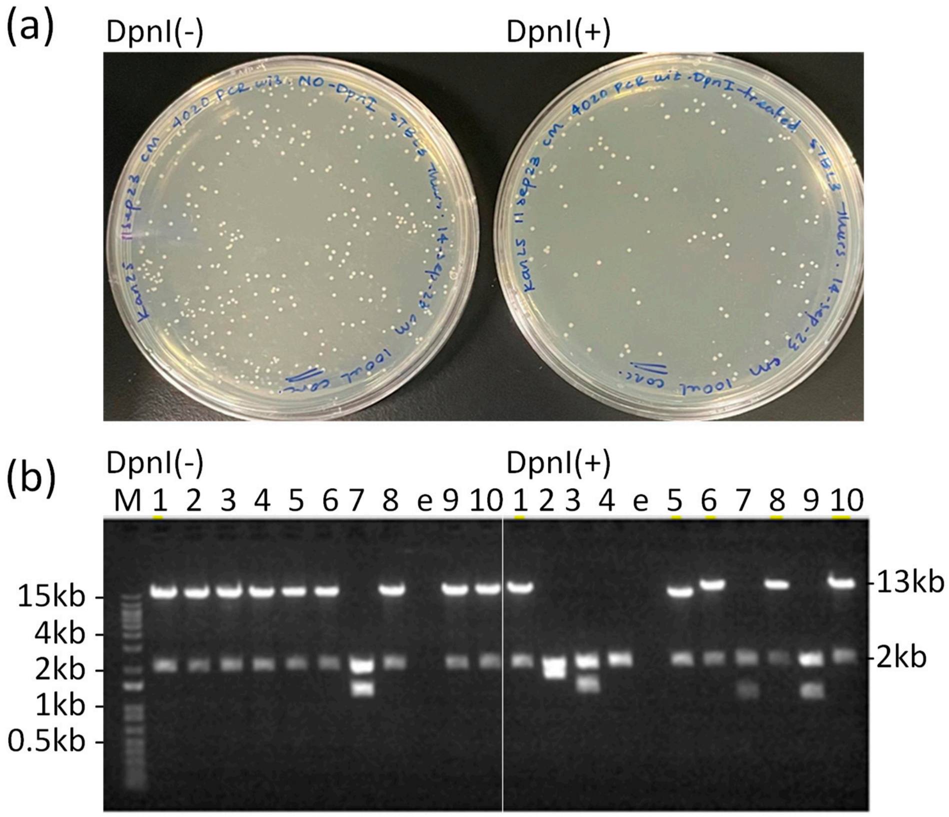


**Figure S2.** Characterization of TAP fragment after high-fidelity, long-range PCR and treatment with DpnI. (a) TAP fragment, untreated with DpnI (left) and treated with DpnI (right) was transformed into Stbl3 *E. coli* cells to compare the number of colonies resulting from residual pMG4020 plasmid template in a high-fidelity PCR reaction. (b) Plasmid DNA from ten colonies from each plate were isolated and digested with NruI and EcoRV restriction enzymes to identify template pMG4020 plasmid. Expected sizes for pMG4020 are 13kb and 2kb indicated on the right.
